# Society Meetings

**Published:** 1902-05

**Authors:** 


### SOCIETY MEETINGS.

The American Medical Association will hold its fifty-third annual meeting at Saratoga Springs, N. Y., June 10-13, 1902, under the presidency of Dr. John A. Wyeth, of New York. The headquarters of the association will be at the United States hotel.

---

The American Congress of Tuberculosis will hold its next annual session at New York, May 14-16, 1902, under the presidency of Dr. Henry D. Holton, of Brattleboro, Vt. Dr. John H. Pryor, of Buffalo, is one of the vice-presidents. Full details of the arrangements will appear in the *Medico-Legal Journal.*

---

The Mississippi Valley Medical Association will hold its twenty-eight annual meeting at Kansas City, October 15-17, 1902, under the presidency of Dr. S. P. Collings, of Hot Springs, Ark. Titles of papers should be sent at an early date to Dr. H. E. Tuley, 111 Kentucky Street, Louisville.

---

The American Electro-therapeutic Association will hold its twelfth annual meeting, at the Kaaterskill, Catskill Mountains, N. Y., on Tuesday, Wednesday and Thursday, September 2, 3, and 4, 1902. The officers are: President, Fred H. Morse, Melrose, Mass.; secretary, George E. Bill, Harrisburg, Pa.; treasurer, R. J. Nunn, Savannah, Ga.

---

The Medical Society of the Missouri Valley held its semi-annual meeting, at Lincoln, on March 20, 1902, Dr. Richard C. Moore, of Omaha, president, in the chair. Three sessions were held, and sixteen interesting papers read. The *Medical Herald* was again made official journal of the society. The next annual meeting will be held in Sioux City, Iowa, September 18, 1902.

---

THE Buffalo Academy of Medicine held meetings during the month of April, 1902, as follows:

*Section on Surgery.*—Tuesday evening, April 1. Program: Blood pressure observations in surgical cases, Harvey Cushing, Baltimore, Md.; Prostatectomy, with report of case, H. E. Hayd; A new instrument for the treatment of diseases of the prostate, J. H. Dowd.

*Section on Medicine.*—Tuesday evening, April 8. Program: Cerebral syphilis, F. S. Crego; Report of a case of tubercular disease of the mediastinum, F. W. McGuire.

*Section on Pathology.*—Tuesday evening, April 15. Program: The limitations of laboratory methods of diagnosis, Charles F. Martin, adjunct professor of medicine, McGill University, Montreal.

*Section on Ophthalmology, Otology, Laryngology and Rhinology.*—Monday evening, April 21. Program: A report of four acute cases of middle-ear disease with mastoid complications, Max Keiser.

*Section on Obstetrics.*—Tuesday evening, April 22. Program: The prophylaxis of uterine cancer, clinically considered, Lewis S. McMurtry, Louisville, Ky.

*Section on Surgery.*—Monday evening, April 28. Program: Pruritus ani, Geo. L. Brown; The treatment of rotary lateral curvature of the spine, Prescott LeBreton; The treatment of chronic prostatitis by the hot water method, J. H. Dowd.

---

THE Missouri State Medical Association will hold its forty-fifth annual meeting at St. Joseph, May 20-22, 1902, under the presidency of Dr. J. D. Griffith, of Kansas City. A most interesting program has been prepared, and the meeting promises to be one of unusual excellence.

---

THE Niagara Falls Academy of Medicine held its regular monthly meeting April 14, 1902, at the office of Dr. C. G. Leo-Wolf. A paper entitled, The use of electricity in medicine was read by Dr. W. A. Scott and Dr. Leo-Wolf delivered the president's address.

